# Cytogenetic and Yq microdeletion screening studies in infertile males

**DOI:** 10.1186/1755-8166-7-S1-P74

**Published:** 2014-01-21

**Authors:** J Suganya, Kamala Selvaraj, SS Muthaiah, RS Chandra

**Affiliations:** 1 Department of Genetics, Dr. ALM PG. Institute of Basic Medical Sciences, University of Madras, Taramani, Chennai, Tamil Nadu, India; 2 G.G. Hospital, 6-e, Thirumoorthy Nagar, Nungambakkam High Road, Nungambakkam, Chennai, Tamil Nadu, India; 3 Kanmani Fertility Centre, 43, South Usman Road, T Nagar, Chennai, Tamil Nadu, India

## 

Infertility is defined as the inability of a sexually active couple to achieve pregnancy despite unprotected intercourse for a period of greater than 12 months. Infertility affects 15% of the infertile couples and in about half of them, male factor is responsible. It is due to low number, abnormal or immobile sperm production, or blockages that prevent the delivery of sperm. Illnesses, injuries, chronic health problems, lifestyle choices and other factors can also play a role. A total of 130 infertile males (43 azoospermic, 31 asthenospermic, 7 cryptozoospermic, 20 oligoasthenozoospermic, 29 normozoospermic) were analyzed for the presence of chromosomal abnormalities and Y chromosome classical microdeletions in the azoospermia factor (AZF) regions. Multiplex PCR amplification of genomic DNA was performed using two STS markers for each of the three non-overlapping regions, AZFa (sY84, sY86), AZFb (sY127, sY134) and AZFc (sY254, sY255) following standard guidelines. The markers ZFX/ZFY and sY14 (SRY) were included as internal controls.

Two individuals showed Klinefelter syndrome (47,XXY) and three cases exhibited reciprocal translocations involving autosomes - t(3;17)(q26.2;p12), t(1;11)(p22.3;p13) and t(1;10)(q25;q24). Polymorphisms involving the heterochromatic regions of Y and the acrocentric chromosomes were seen in seven patients. The rest of the individuals exhibited a normal chromosomal pattern. The occurrence of chromosomal abnormalities was confined to only the azoospermia category which could reflect the small sample size. A deletion involving all the three AZF regions was detected in one individual while another showed a deletion in AZFb (partial) and AZFc regions. These results indicate a distinctly lower frequency of Y chromosomal microdeletions in the selected group of men with idiopathic infertility. The study needs to be extended using more primer sets defining these loci which play a major role in spermatogenesis.

